# Sagittal balance parameters measurement on cervical spine MR images based on superpixel segmentation

**DOI:** 10.3389/fbioe.2024.1337808

**Published:** 2024-04-12

**Authors:** Yi-Fan Zhong, Yu-Xiang Dai, Shi-Pian Li, Ke-Jia Zhu, Yong-Peng Lin, Yu Ran, Lin Chen, Ye Ruan, Peng-Fei Yu, Lin Li, Wen-Xiong Li, Chuang-Long Xu, Zhi-Tao Sun, Kenneth A. Weber, De-Wei Kong, Feng Yang, Wen-Ping Lin, Jiang Chen, Bo-Lai Chen, Hong Jiang, Ying-Jie Zhou, Bo Sheng, Yong-Jun Wang, Ying-Zhong Tian, Yue-Li Sun

**Affiliations:** ^1^ School of Mechatronic Engineering and Automation, Shanghai University, Shanghai, China; ^2^ Shanghai Key Laboratory of Intelligent Manufacturing and Robotics, Shanghai, China; ^3^ Longhua Hospital, Shanghai University of Traditional Chinese Medicine, Shanghai, China; ^4^ Spine Institute, Shanghai University of Traditional Chinese Medicine, Shanghai, China; ^5^ Key Laboratory of Theory and Therapy of Muscles and Bones, Ministry of Education, Shanghai University of Traditional Chinese Medicine, Shanghai, China; ^6^ Department of Orthopedics, Suzhou TCM Hospital affiliated to Nanjing University of Traditional Chinese Medicine, Suzhou, China; ^7^ State Key Laboratory of Traditional Chinese Medicine Syndrome, The Second Affiliated Hospital of Guangzhou University of Chinese Medicine, Guangzhou, China; ^8^ Department of Orthopedics, Dongzhimen Hospital, Beijing University of Chinese Medicine, Beijing, China; ^9^ School of Life and Science, Beijing University of Chinese Medicine, Beijing, China; ^10^ Department of Orthopedics, Shanghai Municipal Hospital of Traditional Chinese Medicine, Shanghai University of Traditional Chinese Medicine, Shanghai, China; ^11^ Spine Disease Institute, Shenzhen Pingle Orthopedic Hospital, Affiliated Hospital of Guangzhou University of Chinese Medicine, Shenzhen, China; ^12^ Second Department of Spinal Surgery, Luoyang Orthopedic-Traumatological Hospital of Henan Province (Henan Provincial Orthopedic Hospital), Luoyang, China; ^13^ Shaanxi University of Chinese Medicine, Xianyang, China; ^14^ Rehabilitation Center, Ningxia Hui Autonomous Region TCM Hospital and TCM Research Institute, Yinchuan, China; ^15^ Shenzhen Traditional Chinese Medicine Hospital, Shenzhen, China; ^16^ Division of Pain Medicine, Department of Anesthesiology, Perioperative and Pain Medicine, Stanford University School of Medicine, Palo Alto, Santa Clara, CA, United States

**Keywords:** superpixel segmentation, cervical spine, magnetic resonance imaging, sagittal balance parameters, artificial intelligence

## Abstract

**Introduction:** Magnetic Resonance Imaging (MRI) is essential in diagnosing cervical spondylosis, providing detailed visualization of osseous and soft tissue structures in the cervical spine. However, manual measurements hinder the assessment of cervical spine sagittal balance, leading to time-consuming and error-prone processes. This study presents the Pyramid DBSCAN Simple Linear Iterative Cluster (PDB-SLIC), an automated segmentation algorithm for vertebral bodies in T2-weighted MR images, aiming to streamline sagittal balance assessment for spinal surgeons.

**Method:** PDB-SLIC combines the SLIC superpixel segmentation algorithm with DBSCAN clustering and underwent rigorous testing using an extensive dataset of T2-weighted mid-sagittal MR images from 4,258 patients across ten hospitals in China. The efficacy of PDB-SLIC was compared against other algorithms and networks in terms of superpixel segmentation quality and vertebral body segmentation accuracy. Validation included a comparative analysis of manual and automated measurements of cervical sagittal parameters and scrutiny of PDB-SLIC’s measurement stability across diverse hospital settings and MR scanning machines.

**Result:** PDB-SLIC outperforms other algorithms in vertebral body segmentation quality, with high accuracy, recall, and Jaccard index. Minimal error deviation was observed compared to manual measurements, with correlation coefficients exceeding 95%. PDB-SLIC demonstrated commendable performance in processing cervical spine T2-weighted MR images from various hospital settings, MRI machines, and patient demographics.

**Discussion:** The PDB-SLIC algorithm emerges as an accurate, objective, and efficient tool for evaluating cervical spine sagittal balance, providing valuable assistance to spinal surgeons in preoperative assessment, surgical strategy formulation, and prognostic inference. Additionally, it facilitates comprehensive measurement of sagittal balance parameters across diverse patient cohorts, contributing to the establishment of normative standards for cervical spine MR imaging.

## 1 Introduction

As global demographics trend toward an aging population, there has been a corresponding surge in the incidence of age-associated maladies (GBD, 2017 Disease and Injury Incidence and Prevalence Collaborators, 2018). Chief among these are cervical and lumbar degenerative diseases, which are exacerbated by the aging process (Kasamkattil et al., 2022). Cervical spondylosis, a syndrome characterized by a spectrum of symptoms and signs, arises from the degeneration of intervertebral discs in the cervical spine. This degeneration can stimulate or impinge upon surrounding tissue structures such as the spinal cord, nerves, and blood vessels (Theodore, 2020). Globally, over a third of individuals experience mechanical neck pain persisting for more than 3 months, with neck pain ranking fourth among causes of mobility impairments. This highlights the significant health implications of cervical spondylosis on a global scale (Global Burden of Disease Study, 2013 Collaborators, 2015; Hurwitz et al., 2018).

Magnetic resonance imaging (MRI) stands as an indispensable diagnostic and monitoring modality for cervical degenerative diseases, offering unparalleled delineation of both osseous and soft tissue structures of the cervical spine (Wright et al., 2018). This enables comprehensive assessment of structural lesions and their relationship to adjacent neurovascular elements. The precise detection and quantification of these alterations provide valuable insights into disease staging, prognostic potential, and prognosis (Li et al., 2023). Cervical sagittal balance plays a crucial role in cervical alignment, stability, and degenerative cervical spine diseases (Azimi et al., 2021; Scheer et al., 2021). Cervical spine MRI provides detailed information on cervical spine structure and facilitates the derivation of sagittal balance parameters (Boudreau et al., 2021). Although standing cervical spine X-rays are economically feasible and readily available, they are limited to two-dimensional images with suboptimal resolution (Azimi et al., 2021; Boudreau et al., 2021). In contrast, MRI, being non-invasive and free from ionizing radiation risks, provides a more comprehensive evaluation of sagittal balance (Lan et al., 2023). However, supine MRI may not accurately reflect the loading state of the cervical spine in an upright posture, potentially leading to measurement inaccuracies (Boudreau et al., 2021). Despite this, MRI remains indispensable for evaluating cervical spine sagittal balance, aiding in therapeutic decision-making and monitoring treatment outcomes. Manual measurement of sagittal balance parameters requires specialized knowledge and training, proving both time-consuming and subject to inherent subjectivity. In light of these challenges, there is an urgent need for a novel tool capable of accurately measuring sagittal balance parameters in cervical spine MR images in accordance with clinical requirements. Such a tool should provide automatic measurements in large heterogenous dataset, generate precise “centroids” via global segmentation, and ensure precision consistent with clinical realities.

In the domain of semantic segmentation, Convolutional Neural Networks (CNN) demonstrate notable advantages in image processing, leveraging their specialized structure featuring local weight sharing. These networks find broad utility across diverse downstream tasks, encompassing methodologies such as Fully Convoltutional Networks (FCN) (Shelhamer et al., 2017), U-Net (Ronneberger et al., 2015), and DeepLab-v3+ (Chen et al., 2018). While supervised neural networks exhibit remarkable accuracy and performance, their reliance on manually annotated gold standard datasets presents a significant hurdle. Addressing cervical spine-related challenges necessitates the involvement of proficient medical practitioners, entailing substantial investment in time and effort for manual segmentation endeavors. This complexity introduces a formidable barrier to dataset creation. As the field of machine learning progresses, increasingly intricate algorithms have emerged to automate the analysis of cervical spine imaging balance parameters. Techniques such as Support Vector Machines (SVM), decision trees, and linear discriminant analysis have been employed to effectively categorize the surface topology of individuals with scoliosis into mild, moderate, and severe deformities (Ramirez et al., 2006). Notably, Lenke and colleagues have introduced an innovative scoliosis classification system utilizing SVM to differentiate among three distinct types of spinal curvature (Lenke et al., 2003). More recently, Zou and collaborators have devised a supervised network, VLTENet, which offers precise determinations of the Cobb angle. However, this method relies on manual segmentation by medical professionals, rendering it both time-consuming and labor-intensive (Zou et al., 2023). Additionally, Amin and colleagues have created an EfficientNet model based on ConvNet architecture, exhibiting significant capability in identifying and classifying spinal curves (Amin et al., 2022). However, its effectiveness is somewhat constrained by a small dataset, potentially limiting its generalizability. In summary, despite notable advancements in the automatic evaluation of cervical spine imaging balance parameters, the field encounters inherent limitations. There is a pressing need for further research focused on developing novel methodologies that integrate unsupervised analysis, enhanced generalization, and improved precision in the autonomous assessment of cervical spine balance parameters.

Several methodologies exist for measuring cervical curvature (Vrtovec et al., 2009). The Harrison method effectively captures variations in segmental curvature while considering local changes in curvature, particularly valuable in biological research concerning vertebral rotation. However, its complexity renders it unsuitable for automation (Harrison et al., 2000). Refshauge et al. adopted a vertebral centroids approach, focusing on the centroids of the C2, C4, and C7 vertebrae to assess cervical lordosis angle, demonstrating high reproducibility (Refshauge et al., 1994). The Vertebral Centroid Measurement Lumbar Lordosis (CCL) method was initially devised for assessing lumbar curvature. This method entails drawing lines between the centroids of the first two lumbar vertebrae and the last two lumbar vertebrae, respectively, with the acute angle formed by these two lines representing the CCL angle. Chen et al. conducted manual evaluations of lumbar curvature using the CCL method, demonstrating its superior reliability in clinical measurements compared to the Cobb method (Chen, 1999). The advantage of the CCL method over the Cobb method lies in its provision of an intuitive understanding of cervical posture while considering in local curvature changes, thereby facilitating early detection of abnormal cervical posture. However, a drawback is encountered in its requirement forvertebrae segmentation and centroids identification from images, a process taking approximately 5–15 min, thus limiting its clinical frequency of use.

In our study, we denoted Chen et al.’s method as the CCL-A method and Refshauge et al.’s as the CCL-B method. Employing both CCL methods, we compared the resulting error and correlations, ultimately selecting the method exhibiting lesser error and greater correlation for measuring cervical curvature.

## 2 Material and methods

### 2.1 Dataset

We conducted a retrospective collection of T2-weighted mid-sagittal cervical spine MRI scans from patients of varying ages across ten hospitals within Mainland China. Subsequently, we segregated tese scans into testing datasets. The participating hospitals comprised Longhua Hospital Affiliated to Shanghai University of Traditional Chinese Medicine, Shanghai Municipal Hospital of Traditional Chinese Medicine, Dongzhimen Hospital Affiliated to Beijing University of Chinese Medicine, The Second Affiliated Hospital of Guangzhou University of Chinese Medicine, Shenzhen Pingle Orthopedic Hospital Affiliated to Guangzhou University of Chinese Medicine, Ningxia Hui Autonomous Region TCM Hospital and TCM Research Institute, Luoyang Orthopedic-Traumatological Hospital Of Henan Province, Affiliated Hospital of Shaanxi University of Chinese Medicine, Shenzhen Traditional Chinese Medicine Hospital, and Suzhou TCM Hospital Affiliated to Nanjing University of Chinese Medicine. Scanning sequences, parameters, and scanners (including strength and vendor) were not standardized across sites to evaluate the generalization of *PDB-SLIC* in a heterogenous, real-world clinical sample.

This study adhered strictly to the principles outlined in the Declaration of Helsinki and received approval from the Institutional Review Board (IRB) of Longhua Hospital Shanghai University of Traditional Chinese Medicine (approval number 2023LCST006). The IRB thoroughly reviewed and approved the research protocol, ensuring the protection of patient privacy and the confidentiality of the clinical data utilized. Given the retrospective nature of the study and the de-identified nature of the data employed, patient consent was waived. All methodologies were conducted in accordance with relevant guidelines and regulations. Prior to analysis, data were anonymized and de-identified uphold patient confidentiality. Furthermore, all researchers involved in data analysis underwent training and adhered to stringent data protection practices.

### 2.2 Preprocess for MR images

**FIGURE 1:**
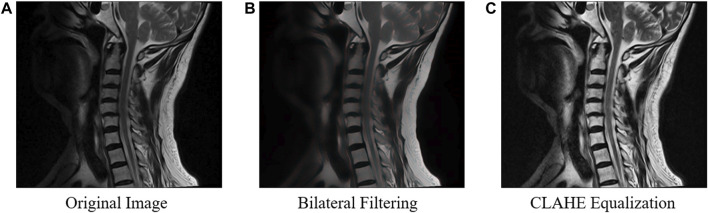
The process of image preprocessing on the original image. To enhance the overall contrast of the original images **(A)**, bilateral filtering **(B)** and CLAHE equalization **(C)** are used in the preprocessing.

To address the issue of inconsistent brightness levels in different images, (Figure 1A) we employed CLAHE equalization to enhance the overall contrast of the images (Figure 1B). And bilateral filtering was used for preprocessing, which effectively addresses issues such as noise interference, unclear image edges, and image blurring, thus further improving the stability of our algorithm (Figure 1C). The calculation formula for bilateral filtering is shown below.

$$G_{\sigma_{s}}\left(∥p-q∥\right)=\exp\;\left(-\frac{{∥p-q∥}^{2}}{2\sigma_{s}^{2}}\right)$$ (1)

$$G_{\sigma_{r}}\left(|I\left(p\right)-I\left(q\right)|\right)=\exp\;\left(-\frac{|I\left(p\right)-I\left(q\right){|}^{2}}{2{\sigma_{r}}^{2}}\right)$$ (2)

$${\bar{I}}_{p}=\frac{1}{W_{p}}\sum_{q\in S}G_{\sigma_{s}}\left(∥p-q∥\right)G_{\sigma_{r}}(|I\left(p\right)-I\left(q\right)|I_{q}$$ (3)

Where $W_{p}=\sum_{q\in S}G_{\sigma_{s}}\left(∥p-q∥\right)G_{\sigma_{r}}\left(|I\left(p\right)-I\left(q\right)|\right)$ is the normalization factor, $G_{\sigma_{s}}$ represents the spatial domain kernel, $G_{\sigma_{r}}$ represents the pixel domain kernel, ${\bar{I}}_{p}$ represents the image after bilateral filtering, $I_{q}$ represents the input image, p 、 q represents the pixel position, $I\left(p\right)$ 、 $I\left(q\right)$ represents the corresponding pixel value.

### 2.3 Superpixel segmentation

Superpixels refer to irregular pixel blocks composed of adjacent pixels with similar texture, color, brightness, and other features that have certain visual significance. By grouping pixels based on the similarity of features between them, a small number of superpixels can be used to represent the characteristics of a large number of pixels, achieving low-complexity and efficient segmentation.

**FIGURE 2:**
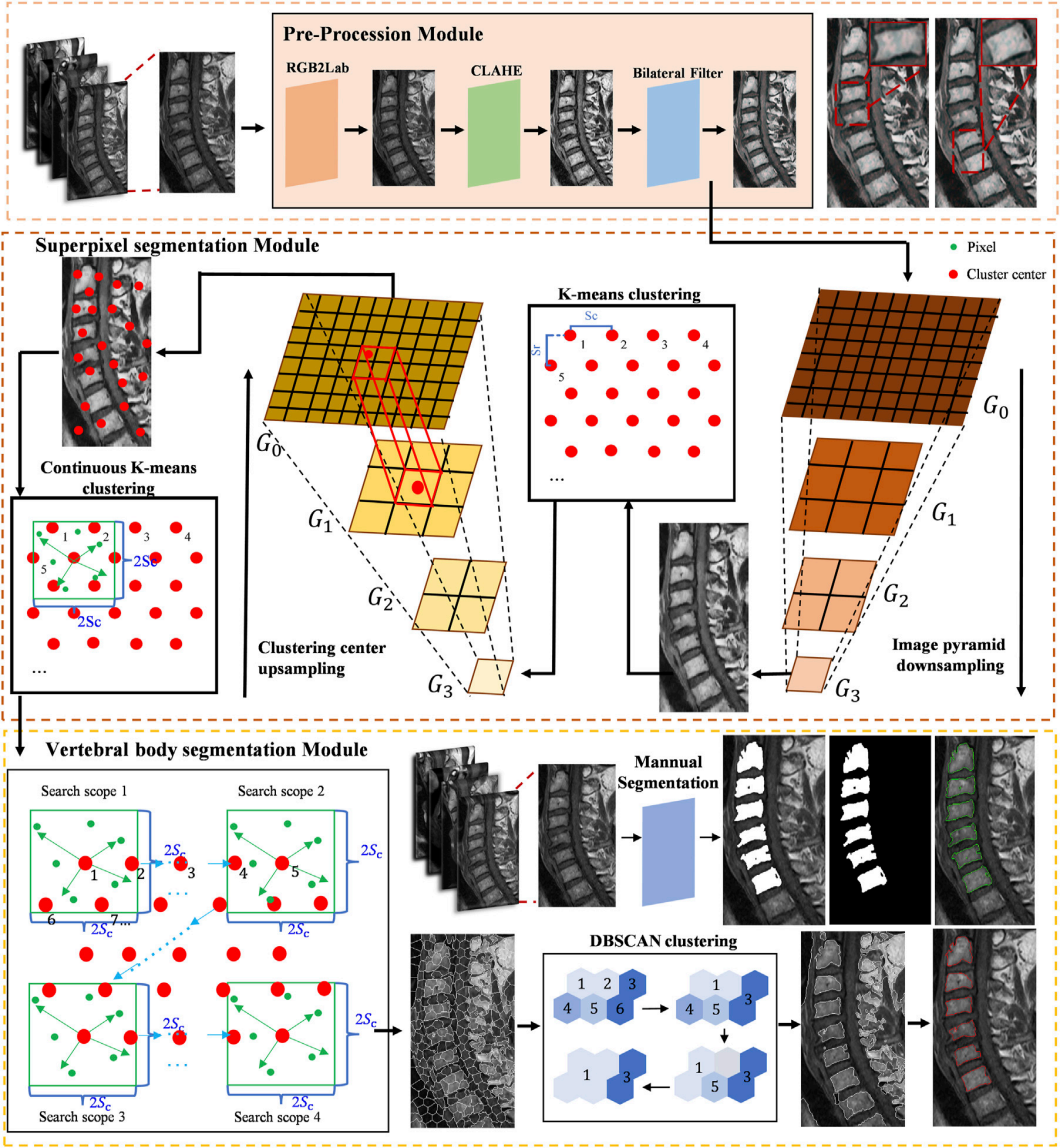
The proposed algorithm flow of cervical curvature measurement based on superpixel segmentation.

The superpixel segmentation method designed in this study generates superpixels through multiple K-means clustering on the image. In order to make the generated superpixels more regular, a distributed hexagonal distribution-based initial clustering center method is designed specifically for vertebral features in this study (Figure 2).

After bilateral filtering, in order to improve the clustering speed and obtain more global image information, a downsampling process is adopted to remove even rows and even columns of the upper-level pyramid image with K-means clustering.

The objective function D is calculated for each clustering center and all pixels within its range. A pixel is assigned to the class whose objective function D with that clustering center is the minimum. The objective function D is defined as follows:

$$d_{c}={\left(L_{j}-L_{i}\right)}^{2}$$ (4)

$$d_{s}={\left(x_{j}-x_{i}\right)}^{2}+{\left(y_{j}-y_{i}\right)}^{2}$$ (5)

$$D=d_{c}+{\left(\frac{m}{S_{c}}\right)}^{2}\times d_{s}$$ (6)

Where m is the weighting factor between brightness and spatial differences. In this experiment, m is set to 15. $S_{c}$ represents the column sampling distance of the clustering centers.

After the initial K-means clustering, the positions and brightness information of the clustering centers are updated using the average values of the position and brightness features of the superpixels, respectively. A new clustering center in the original image corresponds to one updated clustering center in the downsampled image.

**FIGURE 3:**
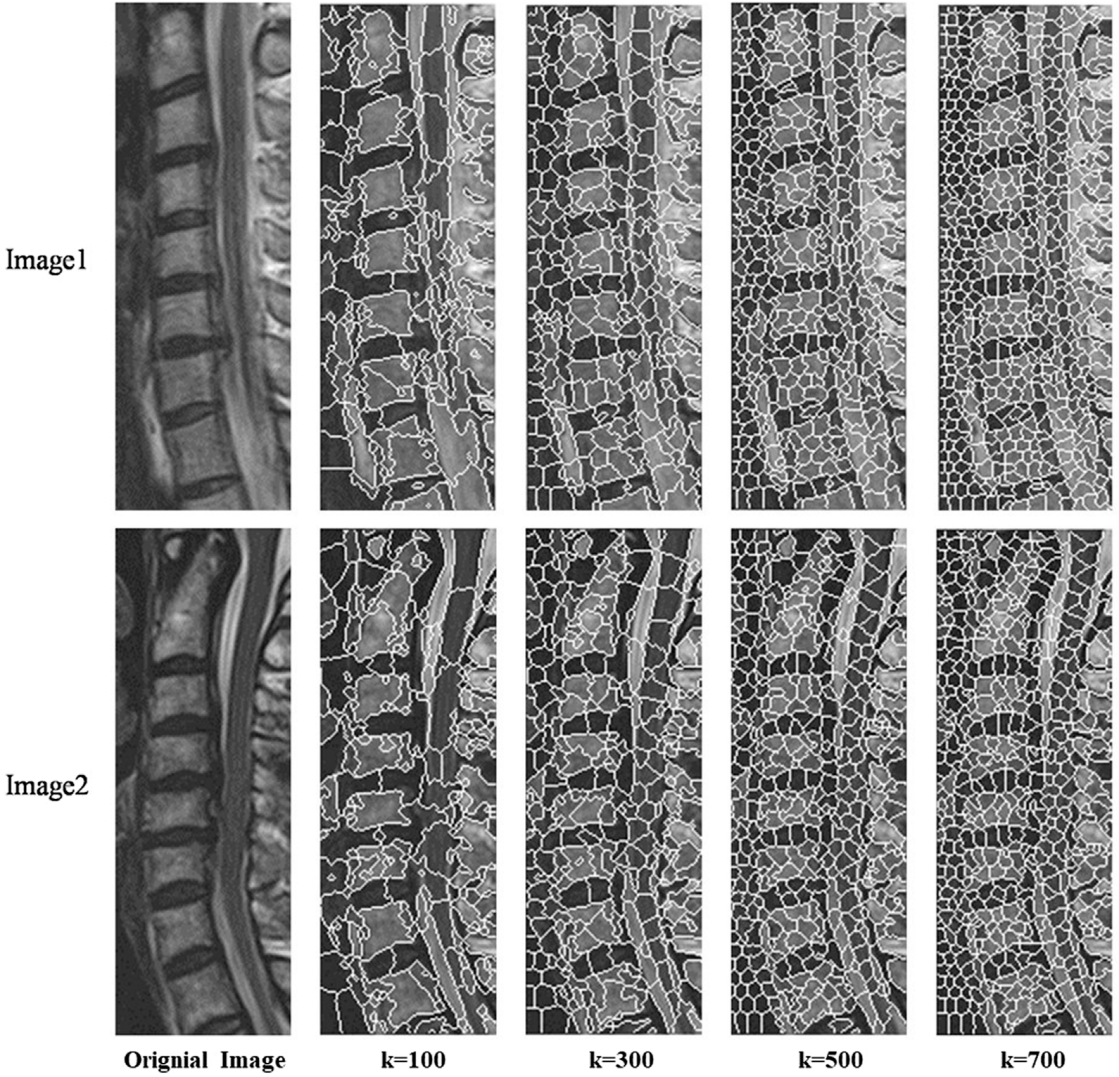
Different superpixel segmentation performance under different number of superpixels.

Next, iterative clustering with the objective function D is performed within their respective ranges based on the new clustering centers. Figure 3 shows the different superpixel segmentation performance under different numbers of superpixels.

The integration of Pyramid-based K-means clustering methodology with multi-resolution images serves to broaden the scope of exploration for clustering centroids, thereby mitigating a constraint inherent in the SLIC algorithm pertaining to its capacity to encapsulate the overarching characteristics of images. Such augmentation markedly amplifies the algorithm’s efficacy in delineating the boundaries of cervical vertebral bodies with heightened precision.

In the stage of superpixel merging, we use the brightness distance metric function Dc to measure the similarity between the seed superpixel and its neighboring superpixels. The definition of the brightness distance metric function Dc is as follows:

$$D_{c}={\left(L_{n}-L_{m}\right)}^{2}$$ (7)

where m represents the seed superpixel, and n represents the neighboring superpixel.

Next, a label set, C, and a candidate set, S, are defined. All superpixels are initially stored in the candidate set. The top-left superpixel of the image is set as the first seed superpixel and assigned a label. The newly formed superpixel resulting from the fusion of the superpixel in the label set with the seed superpixel continues to search for neighboring superpixels within the candidate set until no neighboring superpixels are found in the candidate set. The seed superpixel must be an unlabeled superpixel until all superpixels are labeled, resulting in a complete region segmentation. At this point, doctors can manually select the vertebral regions they consider appropriate to generate the corresponding segmentation results.

### 2.4 Cervical spine curvature and C7 slope measurement

**FIGURE 4:**
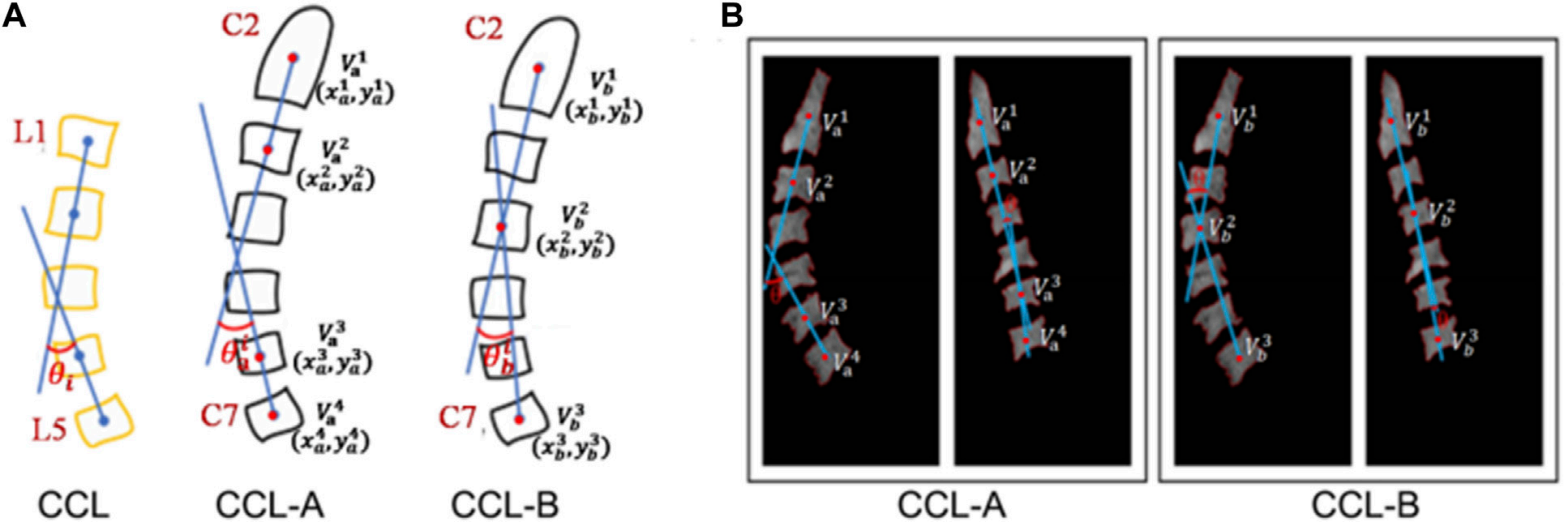
Schematic diagram of cervical curvature measured by CCL methods. **(A)** Modified CCL method was used on the cervical spine (CCL-A and CCL-B). **(B)** The acquisition of the centroid for vertebra and the calculation of curvature in the cervical MRI image.

After obtaining the segmented region of the vertebrae, the vertebral centroid is calculated by the ratio of the horizontal and vertical centroids of the vertebrae to the sum of the pixels in that region (Figure 4). The centroid calculation formula is shown below.

$$x_{a}^{i}=\frac{\sum_{x=\min x_{i}}^{\max{\;x}_{i}}\sum_{y=\min y_{i}}^{\max y_{i}}{x\;*P}_{\text{xy}}}{\sum_{x=\min x_{i}}^{\max{\;x}_{i}}\sum_{y=\min y_{i}}^{\max y_{i}}P_{\text{xy}}}$$ (8)

$$y_{a}^{i}=\frac{\sum_{x=\min x_{i}}^{\max{\;x}_{i}}\sum_{y=\min y_{i}}^{\max y_{i}}{y\;*P}_{\text{xy}}}{\sum_{x=\min x_{i}}^{\max{\;x}_{i}}\sum_{y=\min y_{i}}^{\max y_{i}}P_{\text{xy}}}$$ (9)

Where i is the *i*th vertebra. For CCL-A, i ranges from 1 to 4, while for CCL-B, i ranges from 1 to 3. ${\;x}_{i}$ denotes the set of horizontal coordinates of the *i*th vertebra, $y_{i}$ represents the set of vertical coordinates of the *i*th vertebra, and $P_{\text{xy}}$ represents the pixel value when the height coordinate is x and the width coordinate is y, with $P_{\text{xy}}$ value of 0 or 1.

CCL-A: After obtaining the centroid for each vertebra, the acute angle ( $\theta_{a}$ ) formed by the line connecting the centroids of C2 and C3 and the line connecting the centroids of C6 and C7 is calculated.

CCL-B: After obtaining the centroid for each vertebra, the acute angle ( $\theta_{b}$ ) formed by the line connecting the centroids of C2 and C4 and the line connecting the centroids of C4 and C7 is calculated.

**FIGURE 5:**
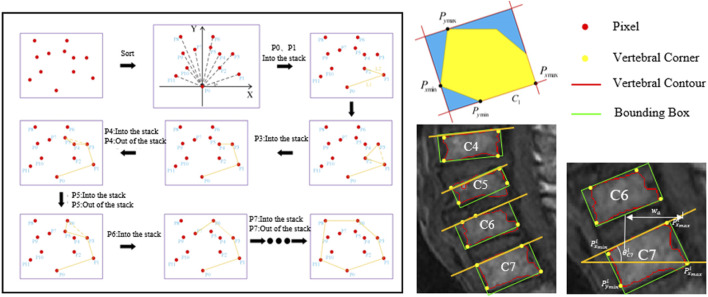
Schematic diagram of C7 slope measured by the minimum bounding rectangle fitting methods.

We also propose a calculation method based on the minimum bounding rectangle fitting to achieve efficient and accurate automatic extraction of the tangent line on the upper edge of the vertebra and measure the corresponding slope. By sequentially locating the pixels in the segmented region of the vertebra to determine four extreme points, the minimum perimeter fitting rectangle is obtained using the rotating calipers method. The upper edge of the rectangle is approximated as the tangent line on the upper edge of the vertebra. The calculation formula for C7 slope is shown below (Figure 5).

$$\theta_{\text{slope}}^{i}=\arccos\;\left(\frac{w_{a}}{\left|\vec{P_{x_{\min}}^{i}P_{x_{\max}}^{i}}\right|}\right)\times \frac{180}{\pi }$$ (10)

Where i represents the *i*th vertebra, with i being 6. $P_{x_{\min}}^{i}$ and $P_{x_{\max}}^{i}$ represent the pixels corresponding to the minimum and maximum horizontal coordinates in the segmented region of the vertebra. $w_{a}$ represents the horizontal distance between these two points.

### 2.5 Segmentation performance evaluation

One of the most important requirements for superpixels is to preserve adherence to object boundaries (Yuan et al., 2021). In order to quantitatively compare the performance of SLIC(Achanta et al., 2012), VBseg (Barbieri et al., 2015), and *PDB-SLIC* in vertebral segmentation quality across different numbers of superpixels, precision, recall, and the Jaccard index are utilized to assess the similarity between algorithmic segmentation and true segmentation of the vertebral region. Additionally, the segmentation performance is compared with classical deep learning networks such as FCN (Shelhamer et al., 2017), DeeplabV3 (Chen et al., 2017), and U-Net (Ronneberger et al., 2015) to identify the best segmentation method.

The comparison of performance parameters among disparate algorithms across numerous superpixel counts, utilizing one-way analysis of variance (ANOVA). The test results are initially subjected to the Shapiro-Wilk test for normality and the Levene’s test for homogeneity of variances, the outcomes of which dictate the appropriate method for variance analysis.

### 2.6 Accuracy validation in sagittal cervical parameters automatic measurement

To compare the performance of the semi-automatic measurement method with experienced spinal surgeons, a subset was randomly extracted from the cervical spine MRI dataset, consisting of 464 T2-weighted cervical MR images. Two spinal surgeons with 5–15 years of clinical experience were recruited to participate in this performance comparison study. Cervical curvature and C7 slope were manually measured using ImageJ2 software (National Institutes of Health, Bethesda, Maryland, United States, https://imagej.net/software/imagej2/) (Rueden et al., 2017). Each image was measured twice independently by both physicians, with the average of the measurements used as the final result. The manual results were then compared with the machine measurements for consistency assessment. The consistency between manual measurements and semi-automatic measurements was assessed using intraclass correlation coefficient (ICC) analysis.

### 2.7 Multicenter generalization validation on cervical spine MR images

To validate the performance of *PDB-SLIC* across different hospital settings, MRI devices, and age demographics, we categorized the cervical MRI dataset based on age group. *PDB-SLIC* was utilized to measure cervical curvature, enabling the analysis of parameter distribution trends across various age cohorts. Our focus was primariy on cervical curvature, C7 slope, and their ratio, with correlation analysis conducted across the datasets.

The distribution of cervical curvature was summarized using means ± standard deviations (SD). To assess the effects of different hospitals, MRI machines, and gneders on cervical curvature measurements, we examined data for normality and equal variances. Multivariate ANOVA or Kruskal-Wallis tests were applied accordingly. Additionally, for comparisons among age groups, we employed one-way ANOVA with Welch’s adjustment and Games-Howell *post hoc* analysis. Pearson’s correlation analysis was utilized to evaluate the correlations among cervical curvature, C7 slope, and their ratio.

### 2.8 Statistical methods

IBM SPSS Statistics for Windows, version 26.0 (IBM Corp., Armonk, NY, United States) was applied to complete the statistical analysis. A *p*-value less than 0.05 was considered statistically significant.

## 3 Experiments and results

### 3.1 MRI dataset baseline characteristics

A total of 4,258 cervical spine MR images from ten hospitals were included in the study, with patient ages ranging from 20 to 90 years. The MR manufacturers included GE, Philips, and Siemens, and the MRI field strength was either 1.5T or 3.0T. The baseline characteristics of the cervical spine MRI image dataset are shown in Table 1.

**TABLE 1:** Baseline characteristics of cervical spine MR cohort.

	Test sets		Test sets
Number	4258 (100.00%)	Number	4258 (100.00%)
Hospital		Age	
Longhua Hospital	100 (2.35%)	20–30	712 (16.72%)
Beijing Dongzhimen	353 (8.29%)	31–40	666 (15.64%)
Guangdong province TCM	400 (9.39%)	41–50	646 (15.17%)
Henan provincial orthopedic	321 (7.54%)	51–60	692 (16.25%)
Ningxia TCM	295 (6.93%)	61–70	762 (17.90%)
Shaanxi First TCM	374 (8.78%)	71–80	551 (12.94%)
Shanghai TCM	374 (8.78%)	81–90	229 (5.38%)
Shenzhen Pingle orthopedic	1458 (34.24%)	MRI equipment	
Shenzhen TCM	326 (7.66%)	GE 1.5T	295 (6.93%)
Suzhou TCM	257 (6.04%)	GE 3.0T	726 (17.05%)
Sex		Philips 3.0T	4219 (9.89%)
Female	2126 (49.93%)	Siemens 1.5T	2089 (49.06%)
Male	2132 (50.07%)	Siemens 3.0T	727 (17.07%)

### 3.2 Optimized segmentation on vertebrae

**FIGURE 6:**
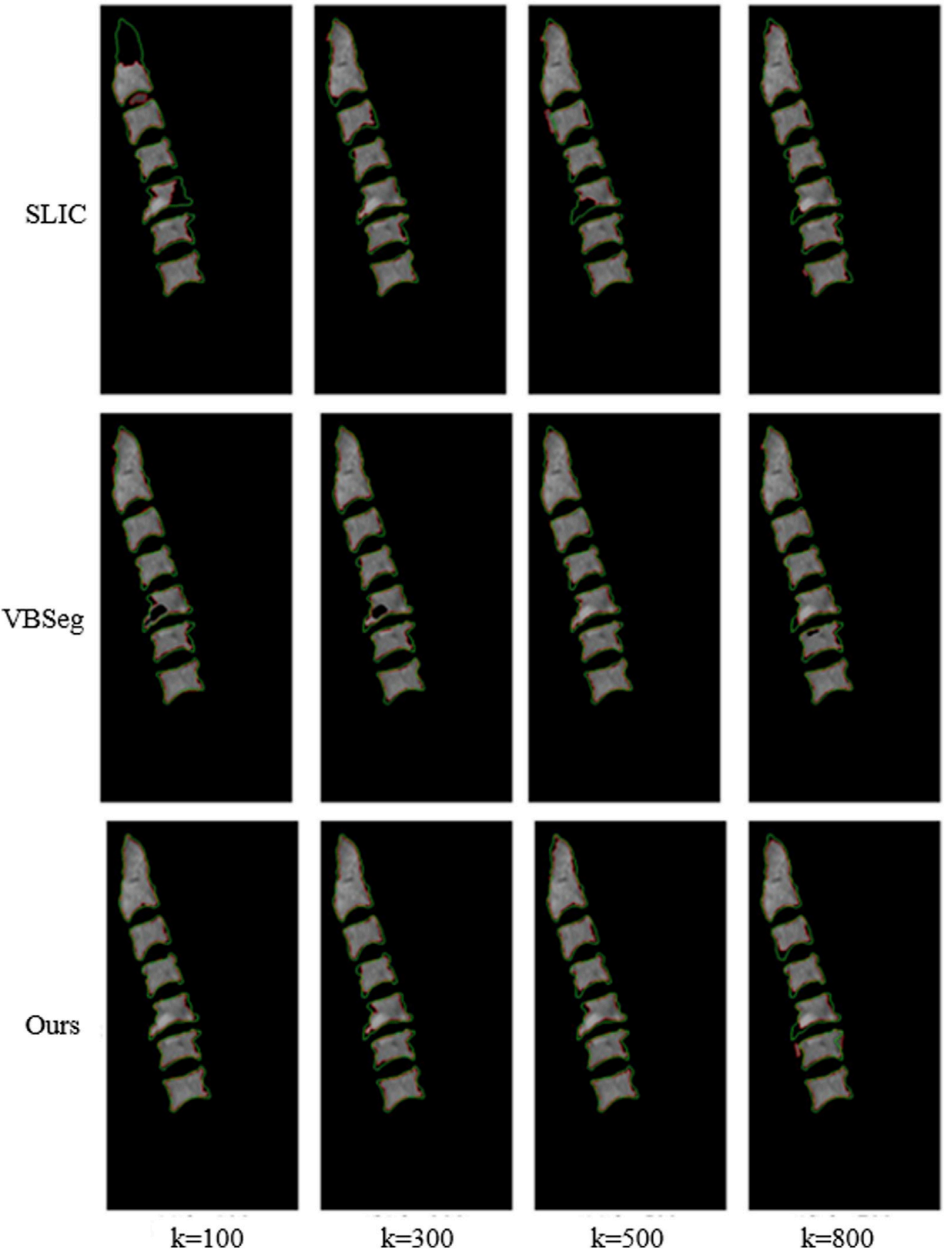
Comparison between SLIC, Vbseg and *PDB-SLIC* algorithm for visual perception of vertebral body segmentation.

Figure 6 illustrated the performance evaluation of the SLIC algorithm, showcasing its inferiority compared to the other algorithms. Notably, significant deficiencies in vertebra segmentation are observed, particularly evident whem the number of superpixels is set to 100, resulting in substantial loss of are in vertebra segmentation. While the Vbseg algorithm achieves superior vertebral body boundary delineation, challenges arise in segmenting local extreme value regions within the vertebrae. This difficulty stems from the algorithm’s utilization of the Otsu method for secondary segmentation of superpixels, leading to enhanced superpixel quality but complicating the segmentation of local extreme value region and subsequent superpixel merging. Consequently, the Vbseg algorithm fails to extract the local extreme value regions within the vertebral body. In contrast, *PDB-SLIC* demonstrates consistent excellence in both the vertebral body region and boundary segmentation, surpassing the performance of the other algorithms overall.

**FIGURE 7:**
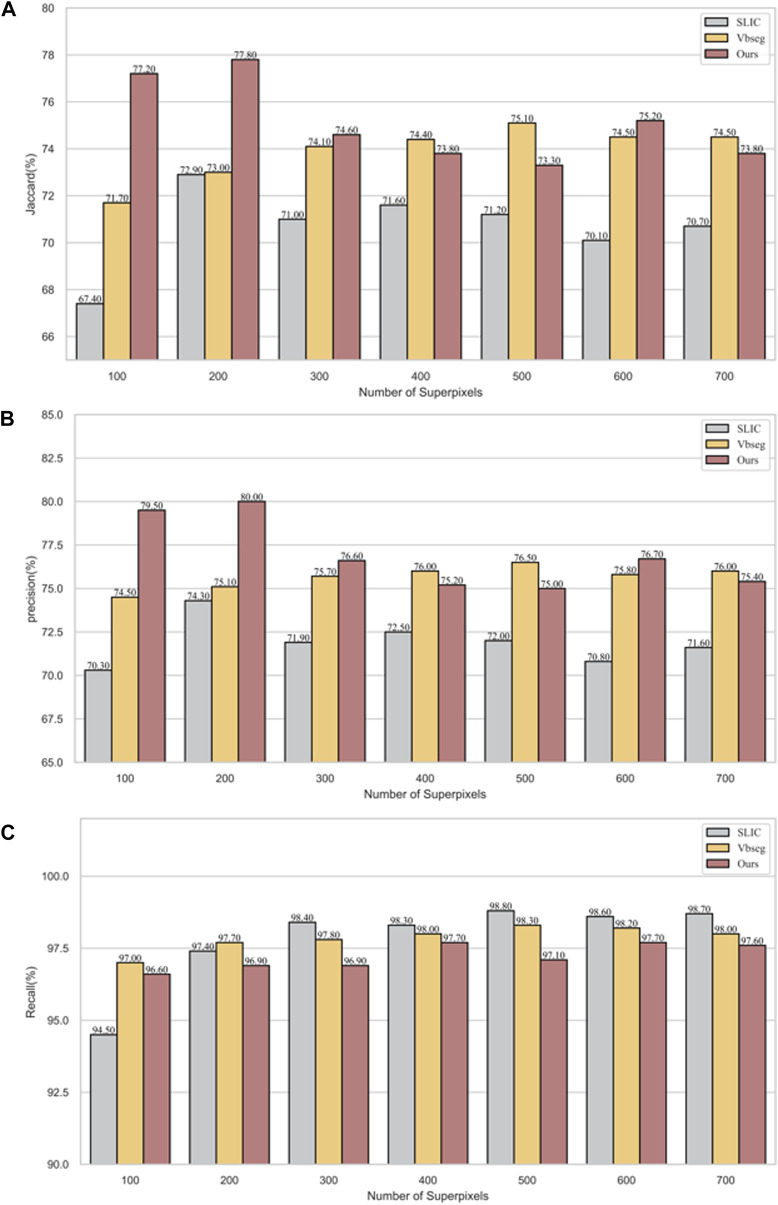
Vertebrae segmentation performance of precision, recall, and Jaccard of SLIC, Vbseg and *PDB-SLIC* algorithm under different superpixel numbers. **(A)** Jaccard; **(B)** Precision; **(C)** Recall.

In Figure 7, precision, recall and Jaccard index evaluation of vertebral body segmentation in cervical MR images are depicted across varying superpixel numbers. A one-way ANOVA, utilizing Fisher’s method, was conducted to analyze the results. The findings reveal that both *PDB-SLIC* and Vbseg algorithms show superior performance compared to the SLIC algorithms in terms of precision (F = 22.517; *p* < 0.001) and Jaccard index (F = 14.842; *p* < 0.001), while the disparity in UE values is statistically insignificant (F = 0.990; *p* = 0.391). Notably, the application of the Otsu method in the Vbseg algorithm contributes to the inability to separate the local extreme value regions within the vertebral body, consequently leading to decreased accuracy and Jaccard index. Upon analysis of the vertebral body segmentation results, both *PDB-SLIC* and SLIC algorithms demonstrate optimal performance when the number of superpixels is set of 200, whereas the Vbseg algorithm performs best with 500 superpixels. Therefore, *PDB-SLIC* exhibits superior vertebrae segmentation performance compared to the other two algorithms.

### 3.3 Segmentation performance validation

Our algorithm presents a significant improvement in accuracy when compared to traditional unsupervised methods, as shown in Table 2. Our results highlight the efficacy of the PDB-SLIC algorithm, showcasing a substantial enhancement of 4.9% in the Jaccard index and 5.7% in precision relative to SLIC. Furthermore, compared to VBSeg, the PDB-SLIC algorithm demonstrates a 2.7% increase in the Jaccard index and a 3.5% improvement in precision. Notably, it even outperforms established supervised neural network algorithms such as FCN and DeeplabV3 networks. The difference in Jaccard value between the PDB-SLIC algorithm and the U-Net network is a mere 0.2%, while the precision value differs by only 0.3%. The variations in recall values are not statistically significant (F = 2.691; *p* = 0.127). These slight disparities compared to classical deep learning networks highlight the exceptional accuracy achieved by our algorithm, eliminating the need for manually annotated datasets by medical professionals.

**TABLE 2:** Segmentation performance validation for different algorithms.

Algorithms	Jaccard ( ± STD )	Precison ( ± STD )	Recall ( ± STD )
STIC	72.9 ± 1.34	74.3 ± 1.64	98.8 ± 1.67
VBSeg	75.1 ± 1.45	76.5 ± 1.63	98.3 ± 1.64
FCN	77.2 ± 1.54	79.6 ± 1.57	98.0 ± 1.73
U-Net	77.6 ± 1.47	79.9 ± 1.56	97.9 ± 1.43
DeeplabV3	77.6 ± 1.55	80.3 ± 1.48	97.6 ± 1.37
PDB-SLIC	77.8 ± 1.44	80.0 ± 1.55	97.7 ± 1.46

### 3.4 Error evaluation in cervical curvature measurements

For the CCL-A method, *PDB-SLIC*’s yielded a mean absolute error of 2.980°, with a standard deviation of 3.167°, and an intraclass correlation coefficient (ICC) of 95.8%. These results closely mirrored those obtained through manual measurements. Conversely, for the CCL-B method, PDB-SLIC exhibited a mean absolute error of 1.604°, with a standard deviation of 1.674°, and an ICC of 97.9%, surpassing the accuracy of manual measurements. These findings indicate the reliability of *PDB-SLIC* in producing measurement results, thereby validating the algorithm’s efficacy and acceptability.

### 3.5 Generalization evaluation of PDB-SLIC by the cervical curvature

**FIGURE 8:**
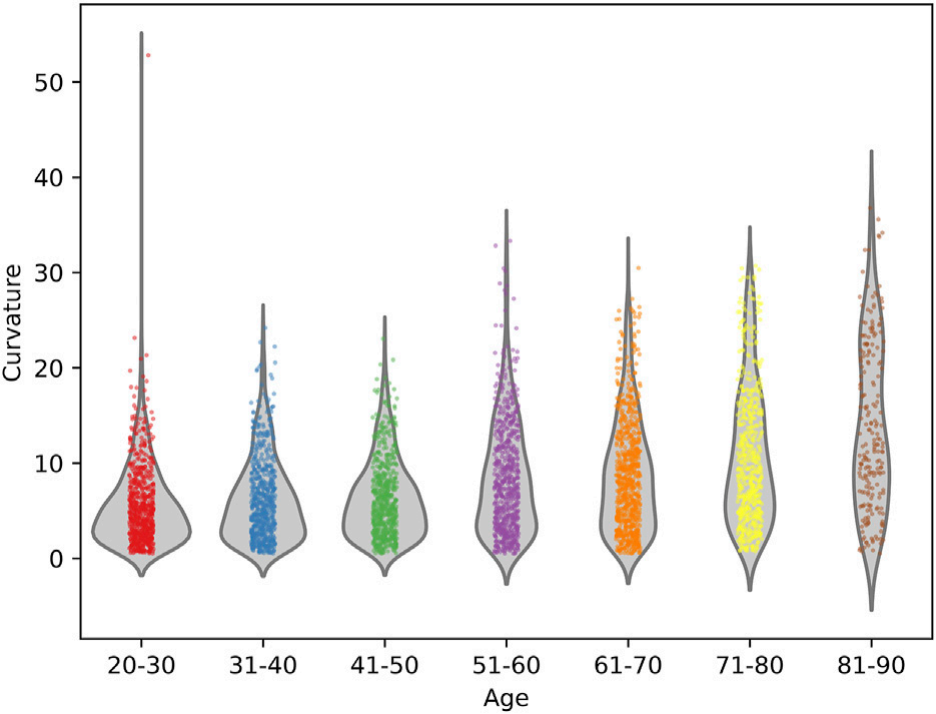
Cervical curvature measurements at different ages group.

*PDB-SLIC* was employed to measure the cervical curvature across the dataset, as summarized in Table 3. A one-way ANOVA, employing Welch’s method, was conducted, followed by a Games-Howell *post hoc* test for pairwise comparisons among groups. The results revealed a significant increases in cervical curvature with age (F = 83.097; *p* < 0.001) (as shown in Figure 8). While no statistical significant differences were observed among the 20–30, 31–40, 41–50, 51–60, and 61–70 age groups (*p* > 0.05), all remaining between-group comparisons yielded statistically significant differences (*p* < 0.05).

**TABLE 3:** Cervical curvature measurements at different ages group.

Dependent	Age	Number	Mean	SD	SE	95% CI
Curvature	20–30	701	5.503	4.285	0.162	5.186–5.820
	31–40	646	6.093	4.328	0.170	5.760–6.427
	41–50	632	6.264	4.111	0.164	5.943–6.584
	51–60	676	8.349	5.872	0.226	7.906–8.792
	61–70	746	8.669	5.877	0.215	8.248–9.091
	71–80	539	10.648	7.141	0.308	10.045–11.251
	81–90	218	13.704	8.711	0.590	12.549–14.861

### 3.6 Error evaluation in cervical curvature measurements

**FIGURE 9:**
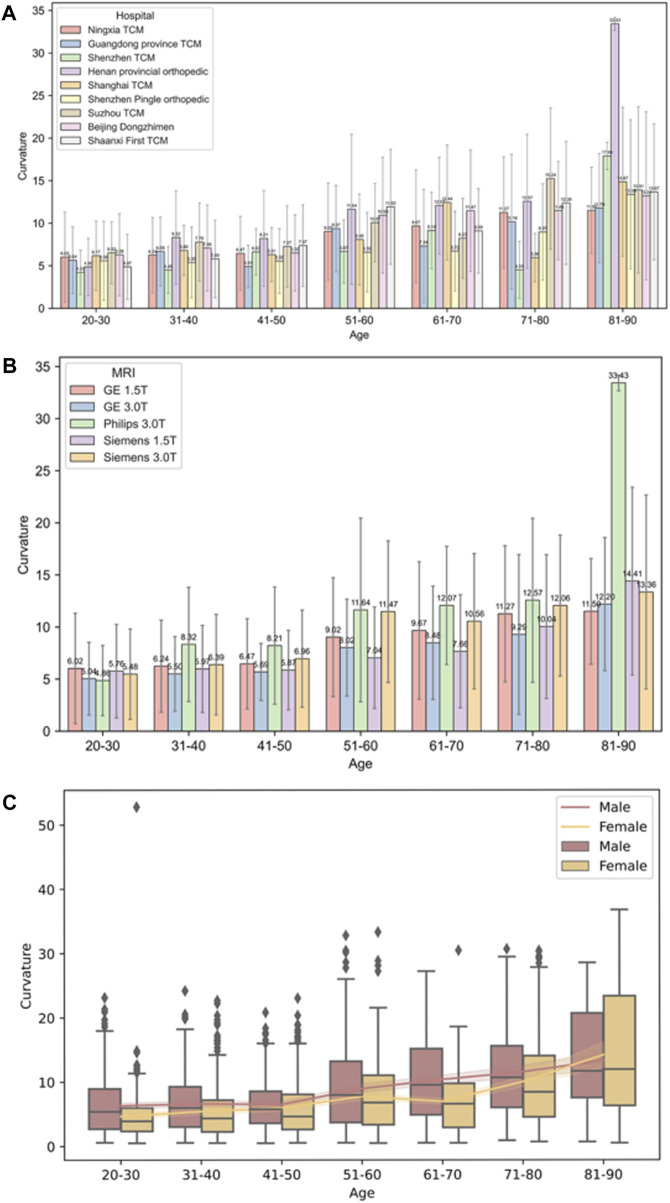
Diagram of trends in cervical curvature across different hospitals, MRI facilities, sex in different age groups. **(A)** Different hospitals; **(B)** Different MRI facilities; **(C)** Different sex.

Non-parametric analysis using the Kruskal-Wallis test was employed to assess the impact of various factors, including different hospitals, MRI machines and genders, on cervical curvature across different age groups. The results showed that there were no statistically significant differences in cervical curvature among different hospitals in the 20–30 and 81–90 age groups (*p* > 0.05), while statistically significant differences were observed in all other age groups (*p* < 0.05); Similarly, in the 20–30, 31–40, and 41–50 age groups, no statistically significant differences were found in the effect of different MRI machines on cervical curvature (*p* > 0.05), whereas significant differences were observed in all other age groups (*p* < 0.05). In the 81–90 age group, sex did not have a statistically significant effect on cervical curvature (*p* > 0.05), while significant differences were noted in all other age groups (*p* < 0.05) (refer to Table 4 and Figure 9).

**TABLE 4:** Kruskal-Wallis test for Curvature in different age groups.

Age groups	Number	Median (IQR)	Hospital		MRI		Sex	
			*H*-value	*p*-value	*H*-value	*p*-value	*H*-value	*p*-value
Total sample	4,158	6.41 (3.28, 10.79)	161.591	<0.001	54.287	<0.001	62.936	<0.001
20–30	701	4.61 (2.51, 7.41)	14.700	0.065	5.645	0.227	23.035	<0.001
31–40	646	5.22 (2.66, 8.35)	43.816	<0.001	7.766	0.101	16.077	<0.001
41–50	632	5.42 (2.97, 8.36)	23.333	0.003	8.325	0.080	6.313	0.012
51–60	676	7.32 (3.53, 11.80)	67.356	<0.001	46.852	<0.001	5.957	0.015
61–70	746	7.77 (3.98, 12.15)	85.919	<0.001	37.536	<0.001	48.513	<0.001
71–80	539	9.08 (4.87, 15.25)	68.662	<0.001	17.542	0.002	7.915	0.005
81–90	218	11.72 (6.82, 21.33)	13.079	0.109	10.328	0.035	0.096	0.757

### 3.7 Correlation analysis between cervical curvature and C7 slope

**FIGURE 10:**
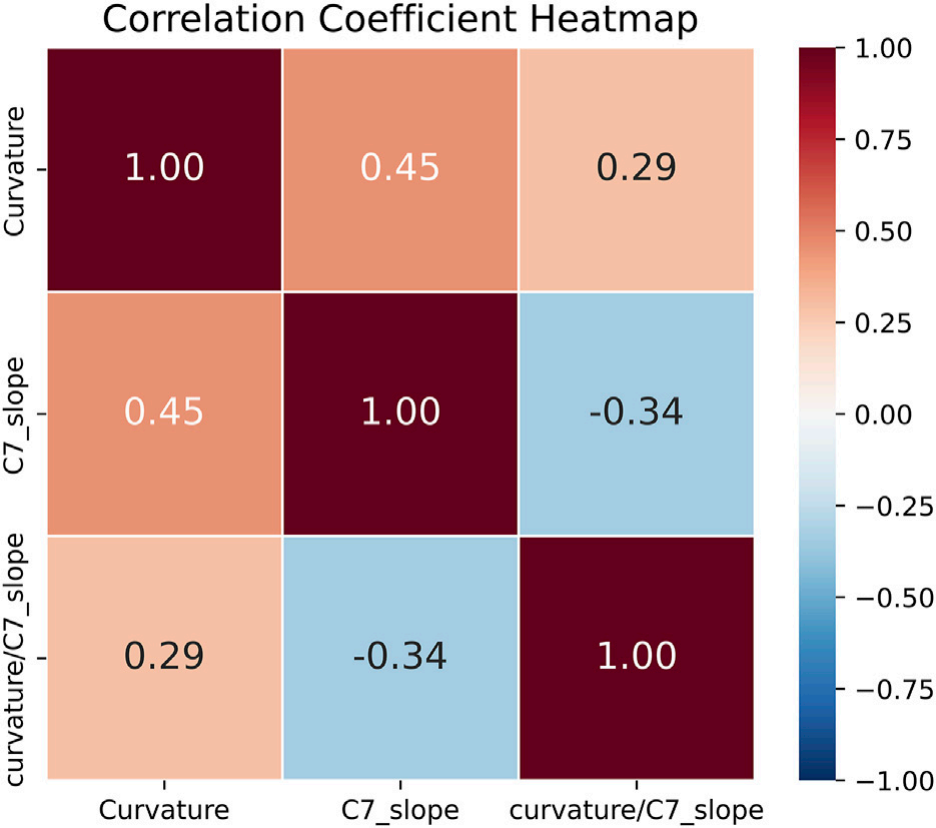
Heatmap of correlation analysis results for three sagittal cervical balance parameters measured by *PDB-SLIC*.

A strong positive correlation was observed between cervical curvature and C7 slope (r = 0.45; 95% CI [0.38, 0.52]; *p* < 0.001). Conversely, the correlation between cervical curvature and the curvature/C7 slope ratio was positive but weaker (r = 0.29; 95% CI [0.21, 0.37]; *p* < 0.001). Additionally, there was a weak negative correlation between C7 slope and the curvature/C7 slope ratio (r = −0.34; 95% CI [−0.42, −0.25]; *p* < 0.001) (as shown in Figure 10).

## 4 Discussion

The current study aims to enhance the measurement of cervical spine curvature to provideclinicians with quantitative insight. While the CCL method and Harrison method are widely acknowledged for their consideration of local curvature changes and high reliability compared to other measurement techniques, their complex operational steps and challenges in achieving automated measurement have limited their exploration in literature. To address this gap and enable semi-automatic measurement of cervical spine curvature using the CCLand Harrison method, this research proposes both a vertebral body segmentation algorithm and a curvature measurement algorithm.

In the preprocessing MR images, bilateral filtering is employed to smooth the images and eliminate noise or strong anisotropic interference (McPhee et al., 2011; Chang and Chang, 2017). Subsequently, a clustering-based superpixel segmentation algorithm is utilized for initial image segmentation. To overcome the limitation of SLIC algorithm in capturing global properties, a novel approach involving superpixels generation through K-means clustering in a multi-resolution image is introduced. This method extends the search range of cluster centers without compromising clustering speed, thereby enhancing image segmentation quality. A comparative experiment on cervical spine MR image dataset is conducted to evaluate superpixel segmentation quality. By incorporating brightness and position information, this method yields compact and orderly superpixels adept at capturing neighborhood features, thus enabling facile transformation from pixel-based to superpixel-based methods.

Addressing the traditional superpixel algorithms’ inability to capture global image properties, our study proposes performing K-means clustering on a multi-resolution image. Through the construction of an image pyramid and downsampling of the preprocessed image, a pyramid with varying resolutions is generated, effectively broadenling the search range of clustering centers and facilitating local clustering on a global scale.

Superpixels represent a valuable technique for segmenting cervical spine MR images. However, they often fall short of achieving comprehensive vertebral segmentation, serving merely as an intermediate step in subsequent image processing (Comaniciu and Meer, 2002; Hamarneh and Li, 2009). The primary reason is that clustering centers seek locally similar pixel features, thereby leading to the division of vertebrae into multiple small superpixels (Shen et al., 2016). To address this challenge, we have devised a method based on the DBSCAN clustering concept, which replaces the traditional algorithm’s search for density-reachable points with a search among neighboring superpixels. Additionally, we employ brightness features as the criterion for similarity between neighboring superpixels. Whenever the brightness similarity falls below a predefined threshold, the superpixels are merged. Unlike other superpixel merging techniques, our method does not necessitate manual intervention, it only requires setting an initial brightness similarity threshold.

Given that superpixel segmentation algorithms typically produce smaller-sized superpixels and struggle to achieve genuine vertebral body segmentation, we employ an enhanced DBSCAN-based superpixel merging approach. This technique merges superpixels to generate new regions, thereby accomplishing vertebral body segmentation. Manual annotation of medical images demands the expertise of trained medical practitioners and consumes substantial time and effort. This complexity poses challenges in assembling datasets for supervised neural networks, making the task cumbersome. Our proposed method introduces an unsupervised algorithm that obviates the need for manual annotation as a gold standard.

In contradistinction to classical neural networks, which rely on manually annotated datasets as their benchmark for training, our methodology showcases the ability to directly forecast segmentation outcomes from raw image data. This pioneering approach alleviates limitations imposed by influential features within the original dataset, thereby mitigating their influence on the segmentation’s generalization performance. Notably, our proposed technique attains a superior level of accuracy when juxtaposed with classical neural networks.

Based on the segmentation results of the vertebral bodies, both the CCL method and Harrison method are employed to measure the curvature of the cervical spine. In response to the challenge posed by the automatic detection of the vertebral body’s posterior edge in the Harrison method, this study introduces two improved approaches for measuring cervical spine curvature: the posterior edge tangent method utilizing minimum rectangle fitting and the centroid tangent method employing interpolation. These methodologies facilitate the automated measurement of cervical spine curvature. Experimental validation confirms the efficacy of the proposed algorithms for cervical spine curvature measurement, exhibiting commendable accuracy.

Recent years have witnessed a growing emphasis on understanding the evolving dynamics of sagittal balance parameters and compensatory mechanisms concerning cervical spine health. Aging is often accompanied by notable alterations in cervical spine curvature and C7 slope, reflecting the body’s adaptation to physiological and structural changes over time (Park et al., 2014; Hu et al., 2020; Zárate-Tejero et al., 2023). Notably, the cervical spine curvature and C7 slope play pivotal roles in sagittal balance evaluation (Le Huec et al., 2015; Iyer et al., 2016; Jalai et al., 2016). Age-related degenerative changes influence these parameters, leading to increased kyphotic postures and C7 slope. Variations in cervical lordosis and C7 slope signify the body’s adaptive response to maintain horizontal gaze and overall alignment as individuals age. With advancing age, the compensatory mechanism of the cervical spine become increasingly crucial. Although the cervical spine possesses a natural ability to adapt and respond to biomechanical stress, this capacity tends to diminish with age, potentially resulting in impaired postural balance (Hu et al., 2020; Zárate-Tejero et al., 2023). Consequently, changes in cervical spine curvature and C7 slope emerge as pivotal indicators of the spine’s adaptive capacity, underscoring the importance of comprehensive evaluation and targeted treatment in older populations. Our findings reveal a discernible correlation between cervical curvature and C7 slope, which intensifies with age and collectively contributes to cervical stability. This evidence highlights the imperative for further exploration into the evolving trends of sagittal balance parameters, paricularly cervical curvature and C7 slope. Enhanced comprehension of these changes and the associated compensatory mechanisms can refined our clinical evaluation and intervention strategies, thereby enhancing spinal health management in the elderly individuals. Indeed, future research endeavors should delve into the clinical relevance of various cervical sagittal balance parameters concerning cervical disorders. In addition, investigations into the normal value ranges of these parameters in large-sample cohorts are warranted to validate their impact on clinical outcomes. Such endwavors hold promise for providing invaluable insights to optimize the assessment of cervical disorders and enhance patient care.

## 5 Conclusion

In this study, we integrate superpixel segmentation with superpixel merging techniques to introduce a novel superpixel-based algorithm for cervical spine vertebral segmentation, termed *PDB-SLIC*. We illustrate the utility of *PDB-SLIC* in automatic measurement of sagittal balance parameters of cervical vertebrae. Our algorithm yields commendable results in vertebral segmentation and exhibits robust generalizability. Comparative analysis with manual measurements reveals enhanced accuracy achieved by the automatic quantitative algorithm. Furthermore, *PDB-SLIC* demonstrates consistent performance and stability across diverse settings, encompassing variations in MR image acquisition across different hospitals, MRI devices, and patient demographics.

## Data Availability

The original contributions presented in the study are included in the article/Supplementary Material, further inquiries can be directed to the corresponding authors.
