# Supplementary figures and images for: Sagittal balance parameters measurement on cervical spine MR images based on superpixel segmentation
Source: Front Bioeng Biotechnol. 2024 Apr 12;12:1337808. doi: 10.3389/fbioe.2024.1337808 (PMC11048045; doi:10.3389/fbioe.2024.1337808)

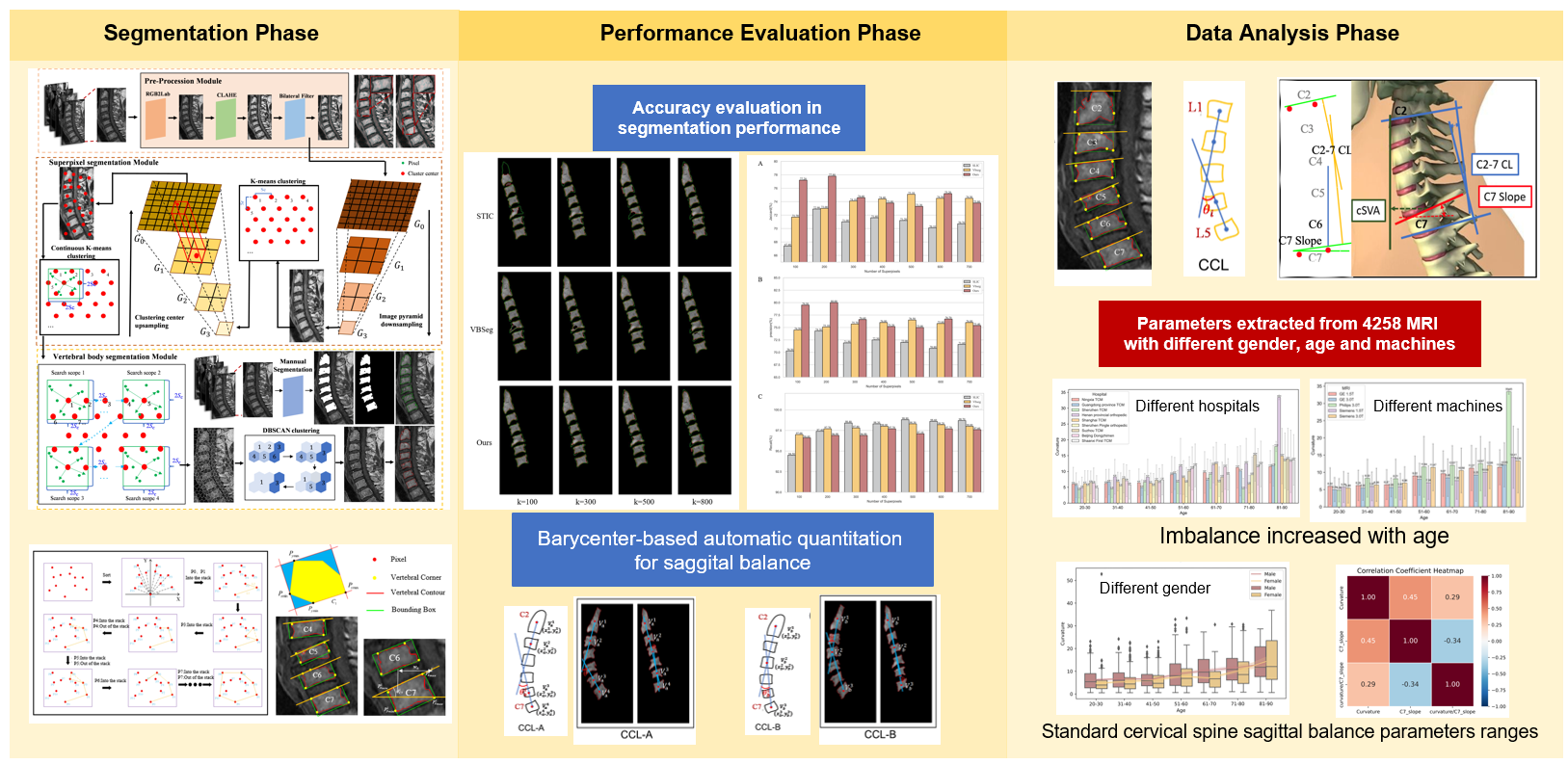

Supplement: Supplementary file 1 [file Image1.TIFF]
